# Concentration of Na^+^-taurocholate-cotransporting polypeptide expressed after in vitro-transcribed mRNA transfection determines susceptibility of hepatoma cells for hepatitis B virus

**DOI:** 10.1038/s41598-021-99263-3

**Published:** 2021-10-05

**Authors:** Andreas Oswald, Anindita Chakraborty, Yi Ni, Jochen M. Wettengel, Stephan Urban, Ulrike Protzer

**Affiliations:** 1 Institute of Virology, School of Medicine, Technical University of Munich/Helmholtz Zentrum München, Munich, Germany; 2 Department of Infectious Diseases, Molecular Virology, University Hospital Heidelberg, Heidelberg, Germany; 3 German Center for Infection Research (DZIF), partner site Heidelberg, Heidelberg, Germany; 4 German Center for Infection Research (DZIF), partner site Munich, Munich, Germany

**Keywords:** Transfection, Hepatitis B

## Abstract

Infection of hepatocytes by hepatitis B virus (HBV) depends on surface expression of its receptor Na^+^-taurocholate-cotransporting polypeptide (NTCP), but sufficient NTCP expression is lacking in most cell lines. NTCP can be introduced by plasmid transfection or transduction by viral vectors to render cells permissive for HBV. However, transient transfection of hepatocyte-derived cell lines is inefficient, resulting in inhomogeneous protein expression and does not allow to adapt the level of NTCP expression. We therefore utilized in vitro transcribed mRNA to introduce NTCP into cells. Optimization using alternative cap structures and nucleotide modifications rendered mRNA transfection into different non-hepatic and hepatic cell lines very efficient. After transfection of mRNA, surface expression and functionality of NTCP was demonstrated by staining with an N-terminal HBV-preS peptide and bile acid uptake. Introduction of NTCP by mRNA transfection increased susceptibility of hepatoma cells to HBV in a dose-dependent manner. Transfection of NTCP mRNA into non-liver cells, in contrast, supported bile acid uptake but did still not render the cells permissive for HBV, demonstrating the requirement for additional host factors. Introduction of candidate host factors by mRNA transfection will allow for fast and convenient analysis of the viral life cycle using a transient, but reliable expression system.

## Introduction

Hepatitis B virus (HBV) harbors the smallest genome amongst DNA viruses with a total length of 3.2. kilobases (kb). The DNA resides inside the viral capsid in form of a partially double-stranded, “relaxed circular” (rc)DNA^1^. Infectious virions are released after envelopment of the capsid together with an excess of “empty” membranous subviral particles, which can be detected as hepatitis B surface antigen (HBsAg).

For viral entry, virions first attach to glycosaminoglycan side chains of cellular surface heparan sulphate proteoglycans (HPSG), followed by specific binding to sodium-dependent taurocholate cotransporting polypeptide (NTCP)^2–4^. NTCP, an integral membrane glycoprotein, is exclusively expressed on hepatocytes and is involved in uptake of glycine/taurine-conjugated bile acids^5,6^. The interaction between NTCP and HBV can be specifically blocked by Myrcludex B (MyrB), a synthetic N-acetylated pre-S1 lipopeptide, that blocks viral entry with high efficacy^7,8^. After internalization of HBV particles, the capsid containing the rcDNA is transported to the nucleus, where the viral genome is released and repaired by cellular enzymes to form a so called covalently closed circular (ccc)DNA^9^. cccDNA serves as transcriptional template for all viral RNAs and as the viral persistence form, that cannot be specially targeted by current antiviral treatment using nucleo(s)tide analogs.

To cure HBV infection and prevent the deadly sequelae of chronic hepatitis B the development of new antiviral therapeutics is required. However, in vitro and in vivo model systems that resemble natural infection are limited. Transient transfection using plasmid DNA (pDNA) or transduction by viral vectors expressing NTCP allow only expression in a proportion of target cells and expression patterns are often inhomogeneous. Stable cell lines or clones are frequently used to study the influence of certain cellular factors. These analyses, however, can be biased due to overexpression of target protein by an exogenous, strong promotor. Inducible systems can limit such effects through time-dependent expression allowing more physiological expression levels. Currently, NTCP overexpressing hepatoma cells are utilized (HuH7, HepG2) because they support productive HBV infection. In such cell lines, overexpression may influence cellular homeostasis^10^. Overexpression of NTCP increases bile acid (BA) uptake, while BA excretion is not possible, resulting in an intracellular accumulation of glycine/taurine-conjugated BAs, sulfated steroids and sulfated thyroid hormones altering cell physiology^11,12^. Moreover, physiological NTCP expression is limited to the basolateral membrane of hepatocytes, whereas overexpression may lead to unphysiological, ubiquitous expression also on apical cell membranes^13^.

Gene delivery using in vitro transcribed (IVT) mRNA could be an interesting alternative, as it allows for rapid expression of a protein of interest in non-differentiated as well as differentiated and even primary cells. IVT mRNA became a promising alternative to pDNA transfection due to its increased transfection efficiency in dividing and non-dividing cells, since mRNA does not need to be delivered into the nucleus of cells during mitosis^14^. Latest improvements, like incorporation of modified nucleosides (5-methyl-CTP (m^5^CTP) and Pseudo-UTP (ψ-UTP), allowed for reduced immunogenicity and higher mRNA stability. Moreover, Kozak sequences introduced in the 5′ untranslated region increased translational capacity and 5′cap-analoga, like the anti-reverse cap analog (ARCA), improved half-life and translational capacity of the RNA^15–20^.

In comparison to viral vectors, IVT mRNAs lack viral proteins or genes that could be reactivated or lead to higher immunogenicity^21,22^. In addition, IVT mRNA transfection allows titration of protein levels to physiological concentrations since protein expression levels can either be adjusted by the amount of mRNA used or by repeated transfection. This has already been utilized in the context of cystic fibrosis, surfactant protein B deficiency in newborns and a lack of erythropoietin in anemia, where an mRNA encoding for the missing protein was delivered into target cells and restored cellular functionality^23–25^. When compared to viral vectors, IVT mRNA has the advantage of neither conferring a risk of insertional mutagenesis nor inducing vector dependent immunity^14,15^.

Since transfection of IVT mRNA offers multiple advantages for hepatoctytes or other hard-to-transfect cell cultures, we applied this technique to analyze the connection between HBV infection and different levels of NTCP expression. We adapted the NTCP level in HepG2 cells, known to support HBV infection in principle, and compared it to HepG2-NTCP-K7 cells overexpressing NTCP^10^. In addition, we used mRNA transfection to generate non-hepatoma cell lines transiently expressing NTCP and analyzed their susceptibility towards HBV infection. We demonstrate that transfection of IVT mRNA encoding for NTCP supports efficient surface expression of functional protein without cytotoxicity in hepatic and non-hepatic cells. Titration of IVT mRNA in HepG2 hepatoma cell lines supported the analysis of dose-dependent effects on cellular function and towards viral infection. However, introduction of NTCP was not sufficient to support HBV infection of non-hepatic cells which most likely requires additional host factors exclusively expressed in hepatocytes.

## Results

### IVT mRNA increases transfection efficiency and protein expression in comparison to pDNA and adenoviral vectors

We first compared IVT mRNA with pDNA or adenoviral vectors expressing NTCP concerning transfection and transduction efficiency as well as subsequent protein expression levels. Therefore, we produced ARCA-capped IVT mRNA stabilized by Ψ-UTP/m^5^CTP modifications that encoded for an NTCP-tdTomato fusion protein. Based on preliminary results (data not shown), the combination of ARCA-cap and Ψ-UTP/m^5^CTP modification maximized mRNA stability as well as protein expression and reduced cytotoxicity^16,26,27^.

**Figure 1:**
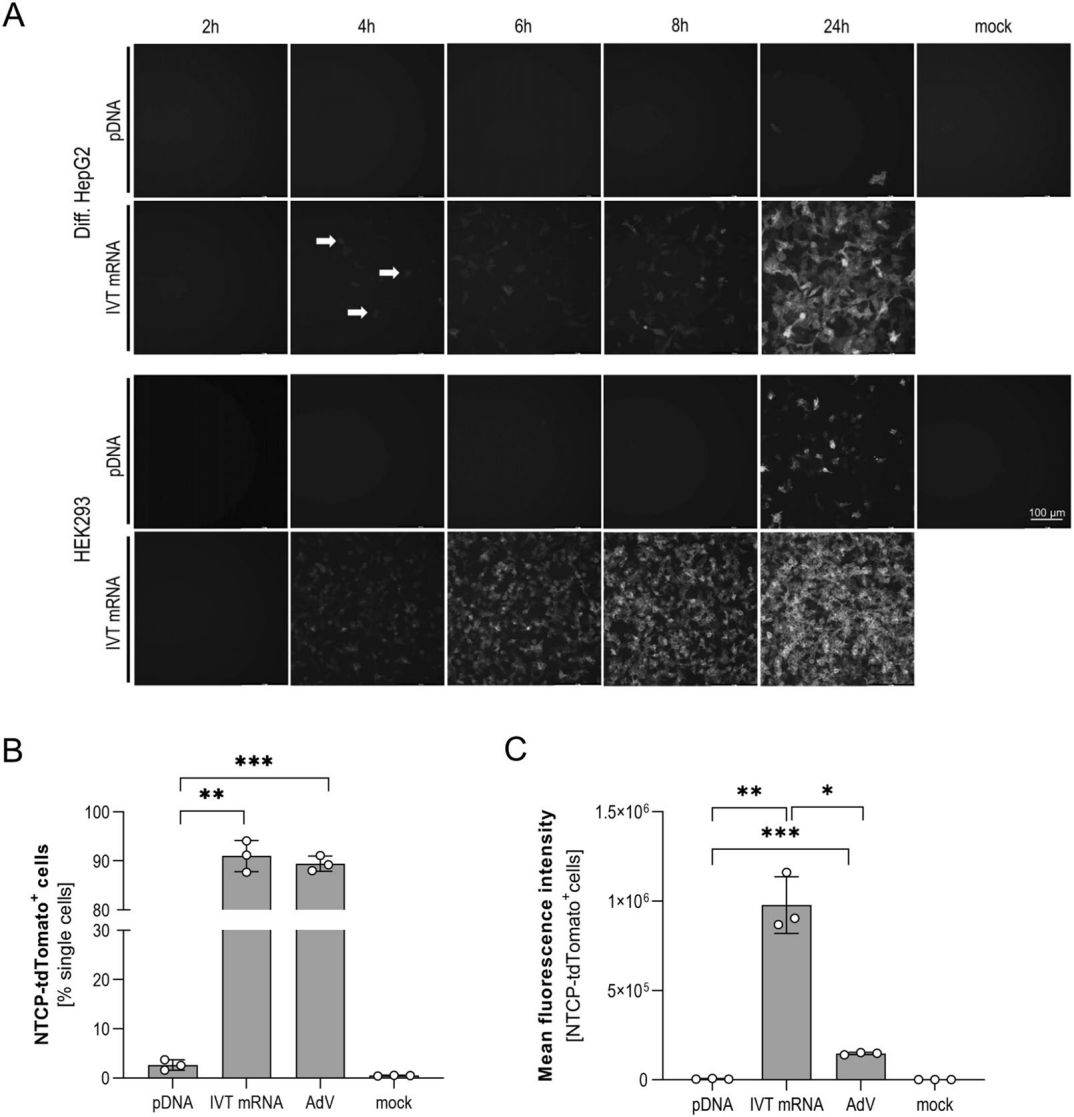
Comparison of IVT mRNA or plasmid transfection or AdV transduction. (**A**) Differentiated HepG2 cells (upper panel) and HEK293 cells (lower panel) were transfected with either 500 ng IVT mRNA or 500 ng pDNA-NTCP-tdTomato. Expression of red fluorescent tdTomato was determined at indicated time points post transfection compared to non-transfected cells (mock) using fluorescence microscopy (TXR channel, 595 nm). Arrows indicate single positive cells; scale bar indicates 100 µm. (**B**,**C**) Flow cytometry analysis (TXR channel, 595 nm) of differentiated HepG2 cells 24 h post transfection of 500 ng IVT mRNA or 500 ng pDNA or infection with AdV-NTCP-tdTomato at MOI of 1 infectious unit/cell. (**B**) Percentage of tdTomato-expressing cells, (**C**) mean fluorescent intensity is given. Mean ± SD of three biological replicates is given. Statistical significance was determined using Welsh corrected Student’s *t* test. (*p < 0.05, **p < 0.01, ***p < 0.001, ****p < 0.0001).

Transfection efficiency of 500 ng IVT mRNA and 500 ng pDNA, both encoding the NTCP-tdTomato fusion protein, was compared in non-dividing HepG2 and HEK293 cells. tdTomato expression was monitored by fluorescence microscopy demonstrating the superior transfection efficacy of IVT mRNA in both cell lines (Fig. 1A)^28^. In both cell lines IVT mRNA transfected cells displayed NTCP expression after 4 h, in contrast to pDNA transfected cells where no positive signal was detected at this time point. One day post transfection efficiency was determined by flow cytometry, showing 90% HepG2 and 95% HEK293 NTCP-tdTomato positive cells when transfected with IVT mRNA. In comparison, pDNA transfection resulted in only 3% HepG2 and 20% HEK293 positive cells. Since adenoviral vectors can more efficiently transduce dividing and non-dividing cells resulting in higher numbers of positive cells^29^, we compared transfection efficiency and protein expression after transfection of IVT mRNA and pDNA with adenoviral vector transduction. We transfected or transduced HepG2 cells with 500 ng IVT mRNA NTCP-tdTomato, 500 ng pDNA-NTCP-tdTomato or adenoviral vector AdV-NTCP-tdTomato at MOI of 1 infectious unit (IU)/cell.

Expression of NTCP-tdTomato was quantified using flow cytometry 24 h post transfection (Fig. 1B). Transfection of IVT mRNA and transduction of adenoviral constructs resulted in higher numbers of positive cells compared to pDNA (91%, 89% and 3%, respectively, Fig. 1C). Calculation of mean fluorescence intensity (MFI) revealed significantly higher expression of NTCP-tdTomato in IVT mRNA compared to adenovirus transduced or pDNA transfected cells.

Taken together, IVT mRNA showed higher transfection efficiency than pDNA and comparable efficiency to that of adenoviral transduction, while resulting in significantly higher protein expression levels. Based on these results we concluded that IVT mRNA offers optimal characteristics to study the infection of HBV in hard-to-transfect hepatoma and other cell lines.

### Transfection of IVT mRNA allows introduction of NTCP into non-hepatic cell lines

**Figure 2:**
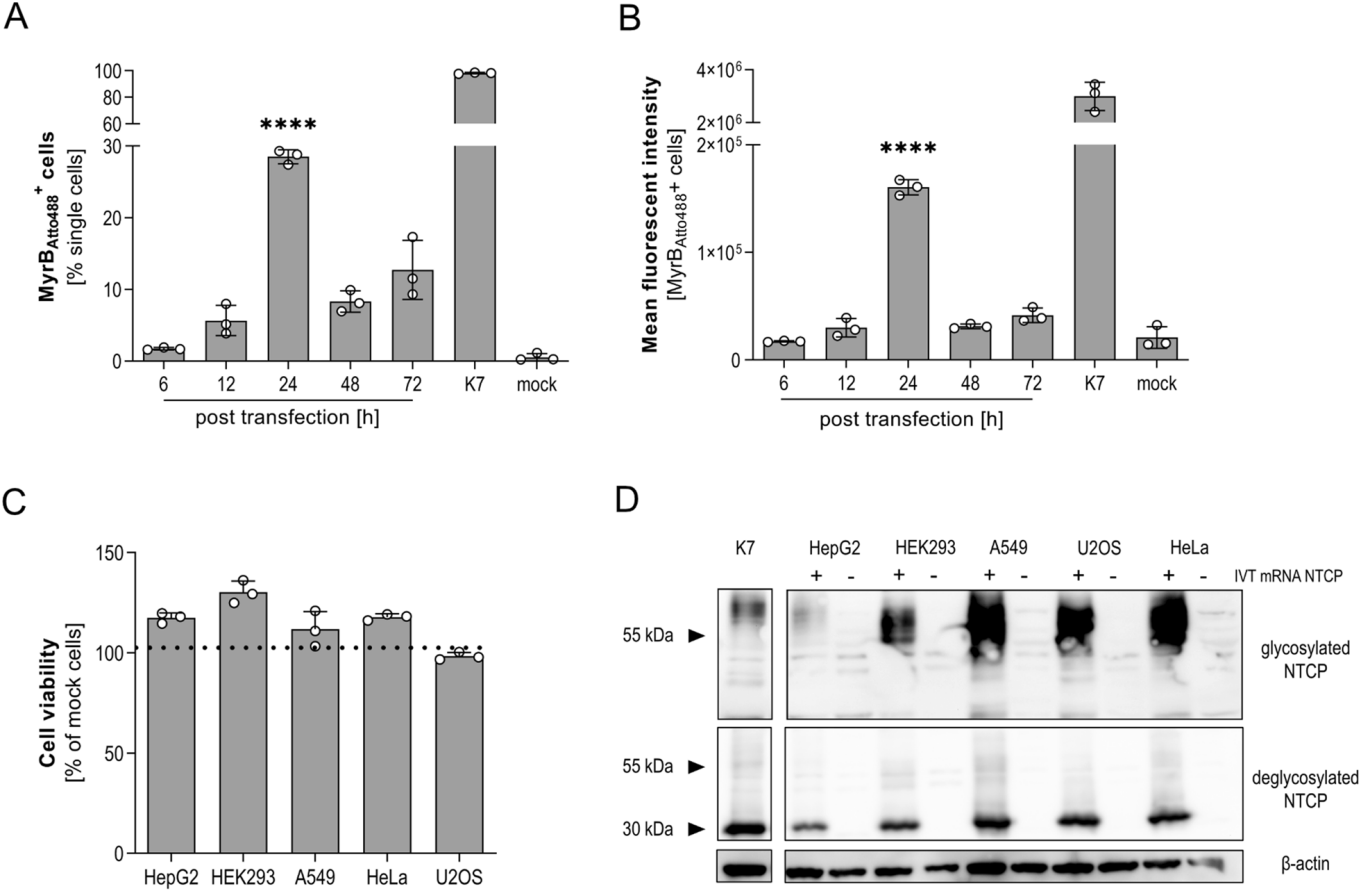
Expression of NTCP in different non-hepatic and hepatic cell lines after IVT mRNA transfection. (**A**) Differentiated HepG2 cells were transfected with 250 ng IVT mRNA expressing NTCP and analyzed by flow cytometry. Total number (left panel) and mean fluorescence intensity of NTCP expression using MyrB_Atto488_ staining (right panel) at indicated time points is given. HepG2-NTCP-K7 (K7) served as positive control. Mean ± SD of three biological replicates is shown. (**B**) 500 ng IVT mRNA NTCP was transfected into HepG2, HEK293, A549, HeLa and U2OS cells. Cytotoxicity was determined via CellTiter Blue assay 24 h post transfection. Mean percentage of viable cells ± SD of three biological replicates normalized to non-transfected control is given. (**C**) Western blot analysis 24 h post transfection of 500 ng IVT mRNA NTCP into respective cell lines. Expression of glycosylated and de-glycosylated proteins was compared. HepG2-NTCP-K7 cell line served as positive, respective parental cells as negative control. Statistical significance was determined using Welsh corrected Student’s *t* test (*p < 0.05, **p < 0.01, ***p < 0.001, ****p < 0.0001).

Due to its half-life of 2–3 days, IVT mRNA transfection is characterized by a fast but transient protein expression^30^. To determine optimal timepoint towards NTCP expression at the cell surface allowing to study its function^26,31^, we transfected HepG2 cells with 250 ng of IVT mRNA encoding for NTCP and monitored protein expression at 6, 12, 24, 48 and 72 h post transfection by flow cytometry. To stain NTCP protein, cells were incubated with 200 nM MyrB_Atto488_^5,7^. As positive control we included HepG2-NTCP-K7 (K7), representing a well characterized cell line for the in vitro investigation of HBV infection^10^. Protein expression levels as well as percentage of NTCP-positive cells reached their maximum 24 h post transfection (Fig. 2A,B).

As NTCP is an essential factor involved in HBV entry, but exclusively expressed on liver cells we aimed at introducing NTCP into non-hepatic cells to determine if this allows productive HBV infection. We transfected 500 ng of IVT mRNA encoding for NTCP into HEK293 (embryonic kidney), A549 (lung carcinoma), HeLa (cervix carcinoma) and U2OS (osteosarcoma) cells. Analysis of cell viability using Cell Titer Blue assay 24 h post transfection revealed no cytotoxicity in any of the transfected cells (Fig. 2C). Western blot analysis demonstrated NTCP expression when cells were transfected with NTCP IVT mRNA at even higher levels than in HepG2 cells (Fig. 2D). Since NTCP is glycosylated, protein lysates were treated with PNGase F to cleave N-glycans and ensure correct glycosylation pattern^5,6^. While HepG2 cells mainly expressed a highly glycosylated form of NTCP, the other cells lines showed more variable glycosylation patterns.

### Transfection of IVT mRNA results in NTCP expression on the cell surface

**Figure 3:**
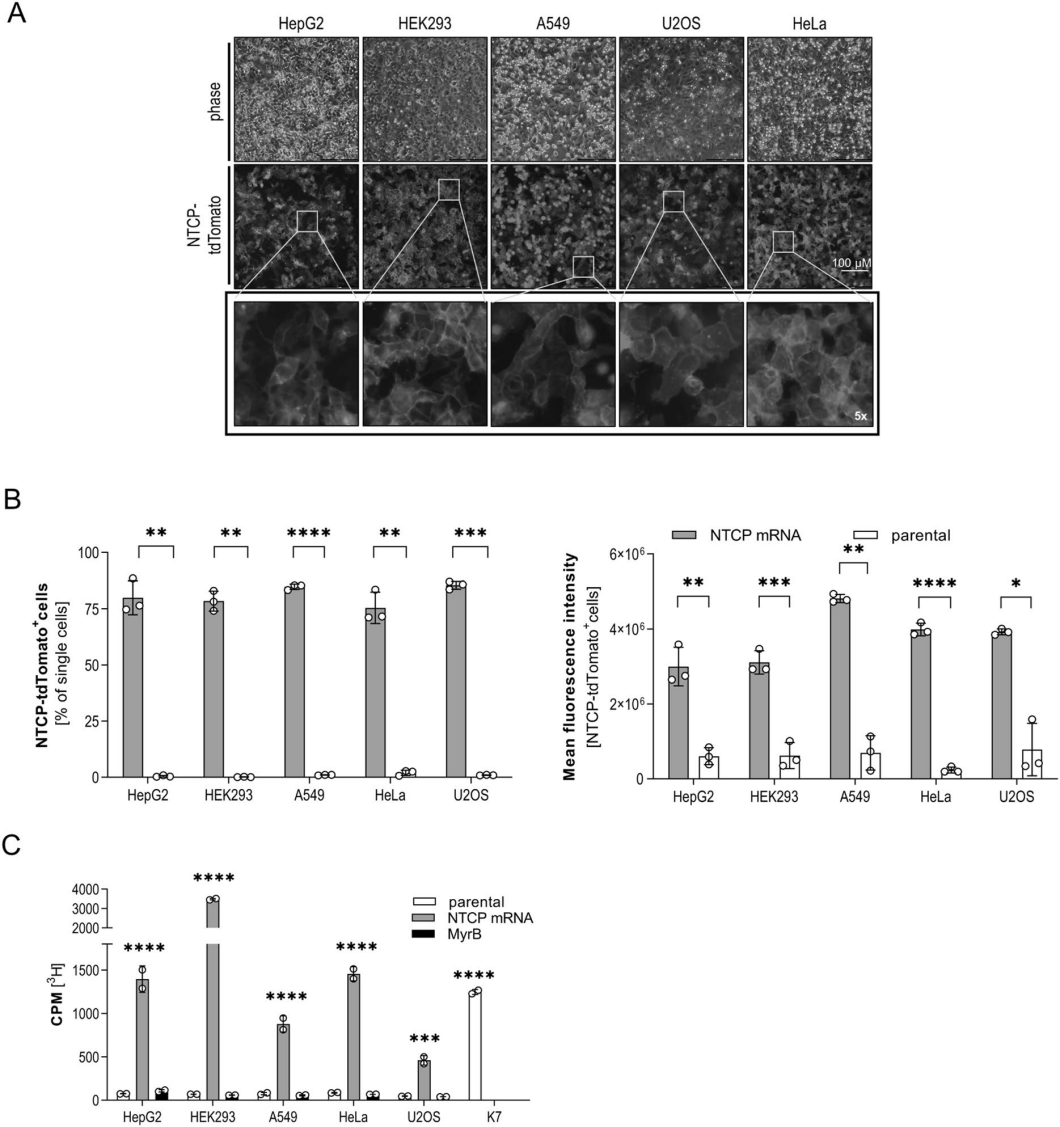
Expression of functional NTCP in non-hepatic cells. 500 ng IVT mRNA encoding NTCP-tdTomato was transfected into HepG2, HEK293, A549, HeLa and U2OS cells. (**A**) Phase contrast (phase) and fluorescence images of indicated cell lines 24 h post transfection (TXR-channel, 595 nm). Enlarged areas on the bottom show fivefold magnification. (**B**) After 24 h, tdTomato fluorescent cells were analyzed by flow cytometry in comparison to parental cells. Percentage of positive cells (left panel) and mean fluorescence intensity (right panels) is given. (**C**) Uptake of taurocholate [^3^H] was determined 24 h post transfection of indicated IVT mRNA transfected cell lines. Parental cells and cells treated with 200 nM MyrB to block uptake served as negative control, HepG2-NTCP-K7 (K7) cells as positive control. Mean ± SD of three biological replicates is shown. Statistical significance was determined using Welsh corrected Student’s *t* test or one-way ANOVA. (*p < 0.05, **p < 0.01, ***p < 0.001, ****p < 0.0001).

To determine whether NTCP correctly localizes to the cell surface, we transfected 500 ng IVT mRNA encoding for NTCP fused to tdTomato. Fluorescence microscopy (Fig. 3A) indicated high transfection efficiency and localization of NTCP-tdTomato to the cell membrane of all cell lines. To quantify expression levels of NTCP-tdTomato and to confirm surface expression of NTCP we used flow cytometry. FACS analysis (Fig. 3B) revealed comparable transfection efficiency of the different cell lines (HepG2: 73.4%; HEK293: 79.8%; A549: 79.1%; HeLa: 75.9%; U2OS: 86.4% tdTomato^+^ cells). Highest protein expression levels were detected in A549 cells as indicated by calculation of the MFI, and all non-hepatic cells showing at least the same level of expression as HepG2 cells. Interestingly, when compared by Western blot, overall expression of NTCP in HepG2 cells was lower but translocation to cell membrane seemed to be more efficient than observed in non-hepatic cells resulting in equivalent surface expression. Next, the physiological function of NTCP protein was determined, using a previously described radioactive labeled taurocholate [^3^H] uptake assay 24 h post transfection. Bile acid uptake is known to be inhibited by MyrB which was included as negative control^7^. Taurocholate [^3^H] internalization (Fig. 3C) was exclusively detected in IVT mRNA transfected cells and was blocked by MyrB treatment. When transfected, cells were compared to the stably NTCP expressing HepG2-NTCP-K7 (K7) cell line, equivalent amounts of bile acids were internalized in HepG2, HeLa and A549 cells. Two-fold higher amounts of bile acids were taken up in HEK293 while lower levels were detected in U2OS cells. These results confirmed surface localization of NTCP in all cells but on the other hand indicated that expression of NTCP is not necessarily associated with localization of functional protein on the cell surface. HepG2 and HepG2-NTCP-K7 expressed lower amounts of NTCP compared to non-hepatic cell lines, but uptake of bile acids was quite comparable. Presumably, transport to the cell membrane is driven by co-factors abundantly present in hepatocytes. In summary, we showed that transfection with IVT mRNA supports the expression of comparable amounts of functional NTCP, allowing bile acid uptake into hepatic and non-hepatic cell lines.

### Transient NTCP expression on non-hepatic cells does not render these permissive for HBV

**Figure 4:**
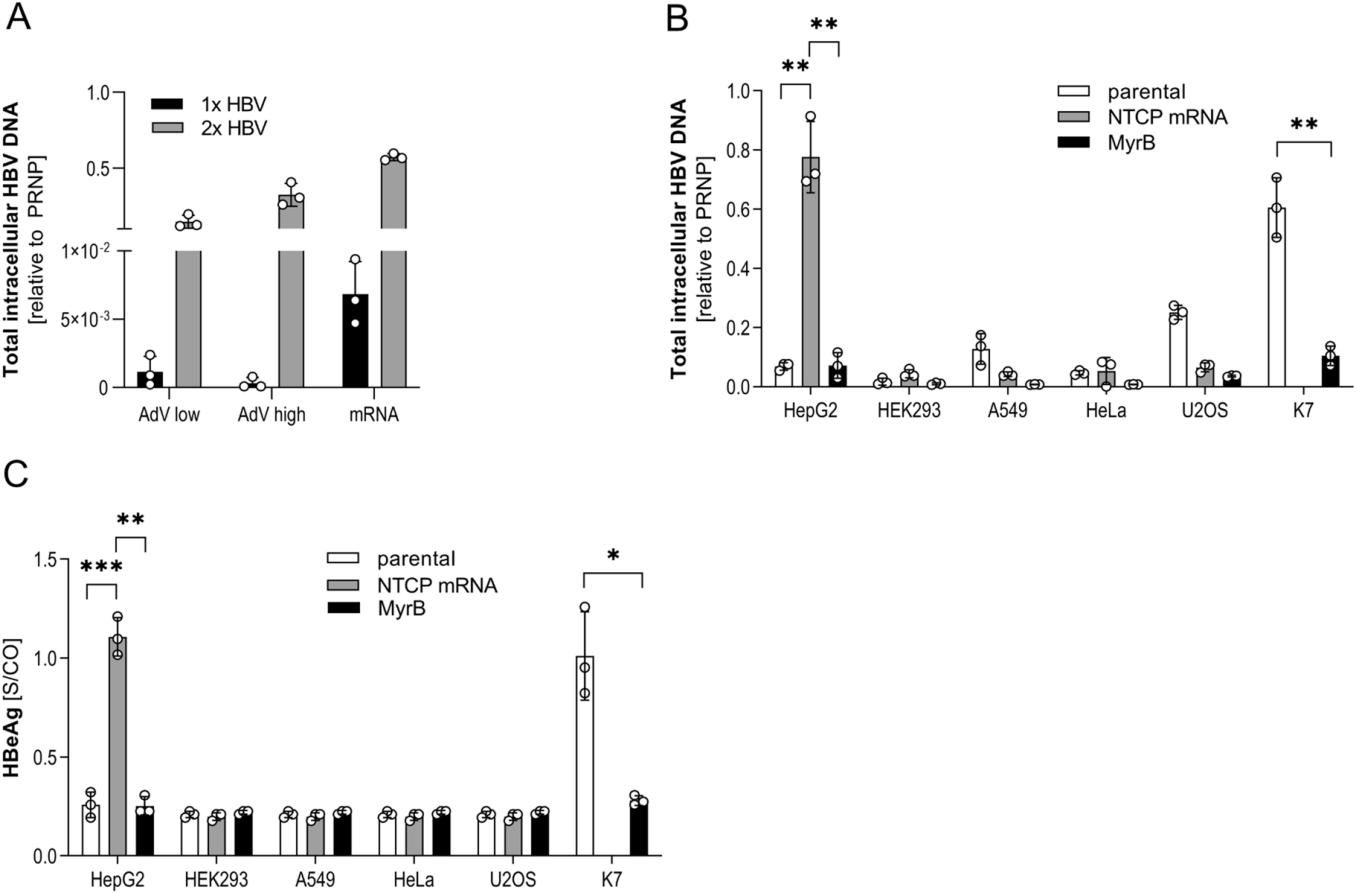
HBV infection in non-hepatic and hepatic cell lines. (**A**) Differentiated HepG2 cells were transfected either with 1000 ng IVT mRNA or transduced with AdV-NTCP at an MOI of 1 or 5 IU/cell, followed by the infection with HBV at an MOI of 200 virions/cell 24 h (1 × HBV) or 24 h and 72 h (2 × HBV) post transfection/transduction. Analysis of total intracellular HBV DNA was performed 7 days post infection. (**B**) 24 h post transfection with IVT mRNA, NTCP-transfected cells were infected with HBV at MOI 200 virions/cell. Quantification of internalized HBV DNA by qPCR relative to human prion protein PRNP 6 h post inoculation. (**C**) HBeAg measurement in cell culture supernatant. HepG2-NTCP-K7 and transfected HepG2 served as positive control. Treatment with 200 nM MyrB before infection to inhibit HBV entry was applied as negative control. In all experiments, mean ± SD of three biological. Statistical significance was determined using Welsh corrected Student’s *t* test or two-way ANOVA. (*p < 0.05, **p < 0.01, ***p < 0.001, ****p < 0.0001).

Next, we investigated whether IVT mRNA transfection would be as efficient as an adenoviral gene transfer to render cells permissive for HBV infection. HepG2 cells were differentiated by the addition of DMSO and either transfected with 1000 ng of NTCP IVT mRNA or AdV-NTCP at an MOI of 1 or 5 IU/cell. Subsequently, cells were infected with HBV at an MOI of 200 virons/cell either after 24 or after 24 and 72 h (Fig. 4A) to give sufficient time for protein expression and translocation. Quantification of intracellular HBV DNA by qPCR demonstrated that IVT mRNA allowed for more efficient infection with HBV after 24 h and 72 h and was thus used to evaluate HBV infection of non-hepatic cell lines.

To determine if non-hepatic cells internalize HBV, we transfected them with 500 ng of NTCP IVT mRNA and monitored HBV uptake by measuring intracellular HBV DNA 6 h post inoculation^32^. Successful infection was determined by detection of HBeAg which is secreted from infected cells after establishment of HBV cccDNA in the nucleus of infected cells. HepG2 cells transiently expressing NTCP took up HBV as efficiently as the stably transfected HepG2-NTCP-K7 clone (Fig. 4B). Taking advantage of the fact that the interaction of HBV and NTCP can be blocked by MyrB^7,8^, we used MyrB to control specificity of HBV infection and showed that virus uptake was inhibited by MyrB (Fig. 4B). Non-hepatic cells in contrast showed no or little (U2OS cells) uptake of HBV compared to MyrB treatment or non-transfected parental controls. HBeAg expression and secretion, indicating successful HBV infection was exclusively detected in transfected HepG2 and HepG2-NTCP-K7 cells (Fig. 4C) 7 days post inoculation with HBV proving that transient expression of NTCP resulted in productive HBV infection. Neither transfected nor parental non-hepatic cells did secrete HBeAg showing that—despite expressing functional NTCP—they did not become permissive for HBV (Fig. 4C). This indicated that either additional host factors are required to render non-hepatic cells permissive for HBV or that cellular restriction factors blocking productive HBV infection are expressed.

### IVT mRNA transfection allows concentration dependent expression of NTCP

**Figure 5:**
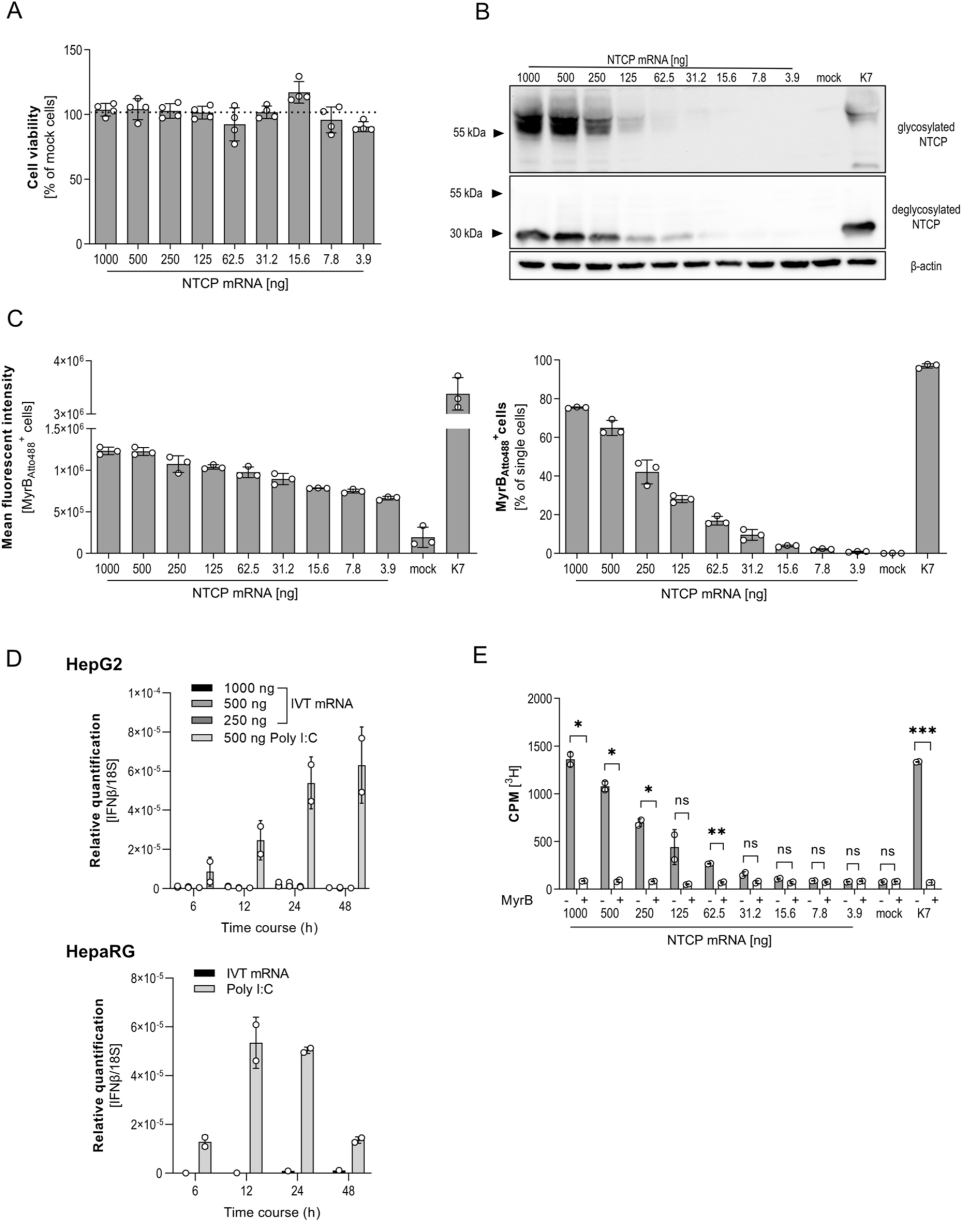
Expression and functionality of NTCP protein dependent on the dose of ICT mRNA. HepG2 cells were transfected with increasing amounts of IVT mRNA as indicated. (**A**) Cell viability was determined via CellTiter Blue assay 24 h post transfection. Non-transfected cells were set to 100%. (**B**) Western blot analysis of NTCP expression compared to HepG2-NTCP-K7 cell line. Glycosylated (~ 50 kDa) and deglycosylated (~ 37 kDa) proteins were analyzed. β-Actin was used as loading control. (**C**) Cells were incubated with MyrB_Atto488_^+^ to analyze binding to NTCP on the cell surface using flow cytometry, showing total NTCP^+^ cells stained with MyrB488 (left) and calculated mean fluorescence intensity of MyrB488^+^ cells. (**D**) Relative quantification of time-dependent IFNβ expression by qPCR after transfection of IVT mRNA and Poly I:C in HepG2 (top) and HepaRG (bottom). (**E**) [^3^H] taurocholate was added 24 h post transfection and internalization was measured using a scintillation counter. Treatment with 200 nM MyrB served as negative control. In all experiments, mean ± SD of biological triplicates (**A**,**C**) or biological duplicates (**D**,**E**) is shown. Statistical significance was determined using Welsh corrected Student’s *t* test (*p < 0.05, **p < 0.01, ***p < 0.001, ****p < 0.0001).

In contrast to overexpression systems, titration of target mRNA should allow adjusting protein expression to different levels. To analyze the impact of different NTCP expression levels on hepatocyte function, we transfected cells with IVT mRNA in concentrations ranging from 3.9 to 1000 ng mRNA per well of a 24 well plate. No cytotoxicity of either mRNA transfection or transient NTCP expression was detected (Fig. 5A). Western blot analysis demonstrated a dose-dependent expression of highly glycosylated NTCP. Transfection of 1000 ng resulted in comparable amounts of NTCP expressed in transfected HepG2 cells and in the stable cell line HepG2-NTCP-K7 (Fig. 5B). Flow cytometry after MyrB_Atto488_ staining confirmed dose dependency of NTCP expression by HepG2 cells and revealed that up to 80% of IVT mRNA transfected cells expressed NTCP on the cell surface. However, the results indicated lower membrane localization in mRNA transfection compared to stable expression (Fig. 5C). To exclude IVT mRNA induced immune responses, which could interfere with NTCP function or subsequent HBV infection, we transfected HepG2 cells with 250, 500 and 1000 ng NTCP IVT mRNA as well as 500 ng poly I:C as positive control. At 6, 12, 24 and 48 h after transfection, we isolated RNA and performed qPCR analysis for IFNβ expression, which is linked to innate immune responses towards RNA. Our results (Fig. 5D, top) showed increasing IFNβ expression over time in poly I:C but not in IVT mRNA transfected cells. We repeated the experiment with either 1000 ng IVT mRNA or poly I:C in differentiated HepaRG, a cell line which has a functional interferon pathway^33^, also demonstrating exclusive upregulation of IFNβ in poly I:C treated cells over time (Fig. 5D, bottom). In summary, our results confirmed previous observations that modified IVT mRNA does not activate intracellular pattern recognition pathways^34^.

Next, we investigated how dose-dependent expression of NTCP influences bile acids uptake as determined by uptake of radioactive labeled [^3^H] taurocholate 24 h post transfection. HepG2 cells transfected with increasing doses of IVT mRNA encoding NTCP allowed a dose dependent internalization of radioactive labeled bile acids (Fig. 5E). According to NTCP expression levels (Fig. 5C, left panel), taurocholate uptake by HepG2 cells transfected with 1000 ng NTCP mRNA was comparable to the stable HepG2-NTCP K7 cell line (Fig. 5C). Uptake of taurocholate by IVT mRNA transfected HepG2 cells was blocked using MyrB, indicating specific and NTCP dependent internalization of [^3^H] taurocholate. Transfection with low amounts of mRNA ranging from 3.2 to 31.2 ng/well did not suffice to induce expression of functional NTCP on the hepatocyte surface supporting taurocholate uptake. Taken together, our results revealed that transfection of increasing concentrations of IVT mRNA led to a dose-dependent expression of fully functional NTCP allowing to fulfill its physiological function, i.e. bile acid uptake, while nucleotide modification prevented RNA pattern recognition.

### Uptake of HBV and productive infection of hepatoma cells depends on the concentration of NTCP

**Figure 6:**
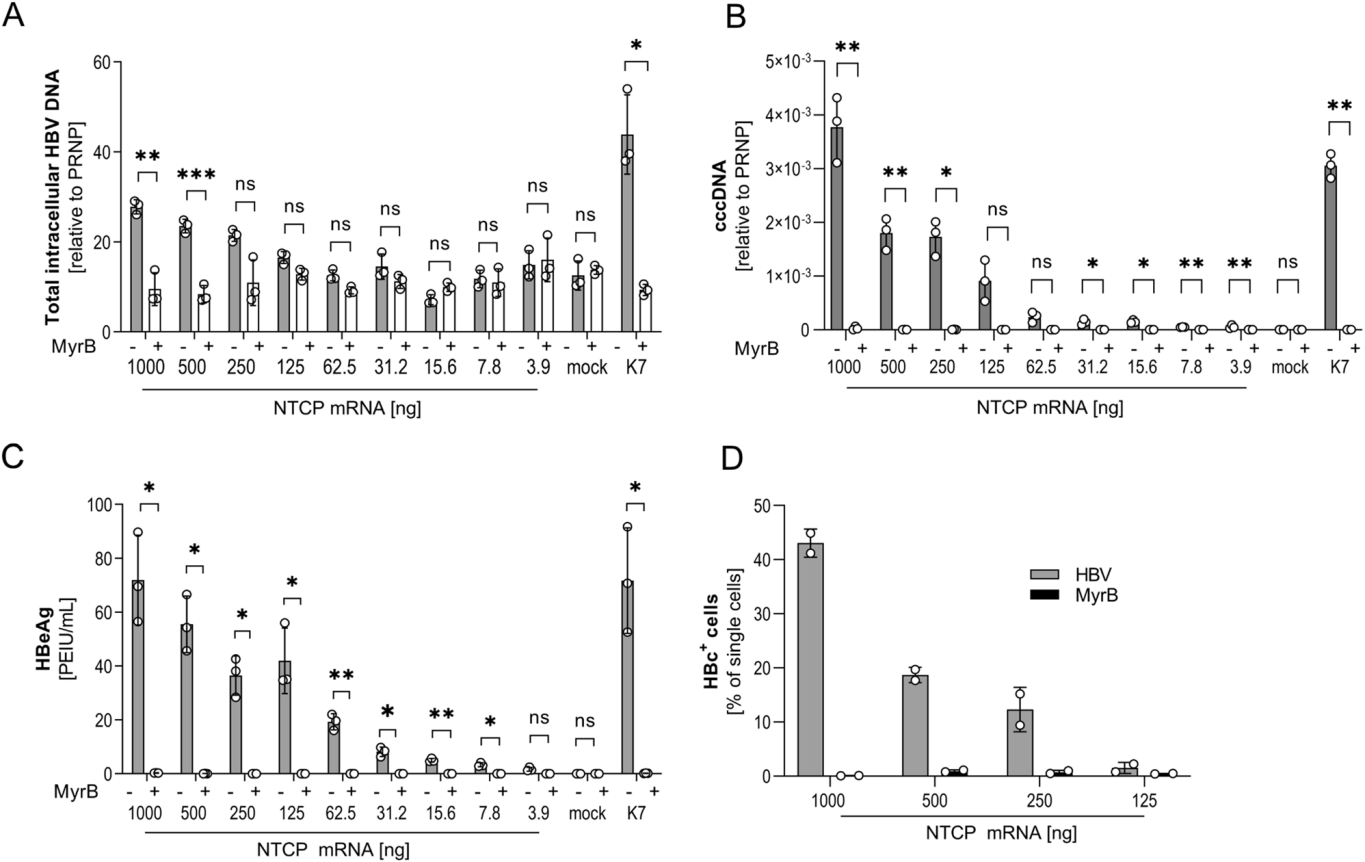
Susceptibility of HepG2 cells for HBV in relation to NTCP expression level. HepG2 cells were transfected with increasing doses of IVT mRNA encoding for NTCP as indicated. (**A**) Quantification of HBV uptake. Total intracellular HBV DNA was measured by qPCR relative to human prion protein PRNP 6 h post inoculation with WT HBV (MOI 200 virions/cell) in transfected HepG2 compared to HepG2-NTCP-K7 cell line. Treatment with 200 nM MyrB served as negative control. (**B**) Quantification of HBeAg secreted into the cell culture medium at 7 days post transfection. (**C**) Quantification of intracellular cccDNA by qPCR relative to human prion protein PRNP 7 days post inoculation. (**D**) To determine HBV infection rates, transfected HepG2 cells were infected with HBV at an MOI of 200 virions/cells and HBV core protein (HBc^+^) expression was analyzed 7 days post infection by flow cytometry. Treatment with 200 nM MyrB served as negative control. In all experiments, mean ± SD of three biological triplicates (**A**–**C**) or biological duplicates (**D**) is shown. Statistical significance was determined using Welsh corrected Student’s *t* test (*p < 0.05, **p < 0.01, ***p < 0.001, ****p < 0.0001).

Finally, we investigated the impact of increasing levels of NTCP for HBV uptake and infection. Differentiated HepG2 cells were transfected with increasing amounts (3.9–1000 ng) of IVT mRNA before HBV was added. The uptake was synchronized by absorption of the virus at 4 °C before cells were shifted to 37 °C to allow internalization. Total intracellular HBV DNA (Fig. 6A) was quantified 6 h later. Results confirmed specific uptake blocked by MyrB treatment when transfecting higher amounts of IVT mRNA. Full HBV infection was analyzed 7 days post infection by HBeAg secretion (Fig. 6B) and cccDNA measurement (Fig. 6C). When ≥ 7.8 ng mRNA per well were transfected, successful HBV infection was detected by HBeAg secretion and establishment of HBV cccDNA in the nucleus of infected cells. As expected, infection could be blocked by MyrB. Although HBV uptake into HepG2 cells transfected with 1000 ng IVT mRNA NTCP was still lower than that into the stable cell line expressing NTCP, efficiency of infection taking cccDNA establishment and HBeAg secretion into account was comparable.

Staining of HBV infection for HBV core protein confirmed dose-dependent infection of IVT mRNA transfected HepG2 cells and demonstrated that MyrB prevented HBV infection specifically even at high IVT mRNA doses (Fig. 6D). Taken together, these results demonstrate that virus uptake and infection of hepatoma cells with HBV strictly depend on the level of NTCP expression.

## Discussion

Factors essential for HBV infection can only recently be studied due to the discovery of the viral receptor, NTCP^3^. We here established the transfection of various cell lines with IVT mRNA as a suitable tool to study whether NTCP expression suffices to render non-hepatic cells permissive for HBV and how NTCP concentration determines the efficiency of HBV infection. IVT mRNA proved to be a powerful alternative to introduce a gene of interest into hard-to-transfect hepatoma cells or primary cells^33^. Thereby, higher transfection efficiency and protein expression levels can be achieved in differentiated HepG2 cells than using plasmid or adenoviral vectors without affecting cell viability or triggering cell-intrinsic immune responses. Our data indicate that the susceptibility of hepatocytes is strictly dependent on the concentration of NTCP expressed and translocated to the cell surface. These findings are in line with a previous study by König et al*.* using a TET-inducible cell culture model^35^, also demonstrating dose- and time-dependent bile acid uptake as well as HBV infection rates. Our study demonstrates the suitability of IVT mRNA transfection for such an approach which is more versatile than TET-inducible systems allowing faster screening in multiple cell lines or primary cells. Additionally, with inducible systems a physiological expression level is difficult to achieve even with time-dependent induction as these systems mostly use strong promotors^35^.

Introduction and overexpression of proteins at comparable levels in different cell lines is an important step to elucidate their cellular function or impact towards permissivity for a pathogen^3,28,36–38^. Currently, viral or plasmid vectors are most frequently used to introduce certain genes into cells facing several restrictions. Efficient pDNA transfection is dependent on the cell type, ranging from high transfection efficiency in HEK293 cells to very low in differentiated or primary cells that do rarely enter the mitotic phase, essential for successful pDNA transfection^28^. In contrast to pDNA, adenoviral or lentiviral vectors allow high transduction efficiency, but may lead to unwanted expression or presentation of vector-derived antigens, genome integration and activation of immune responses^39–41^. Silencing of gene expression by cellular restriction due to bacterial sequences or episomal localization mechanism (e.g. PJA1 and SMC5/6) and inhibition of frequently used viral promotors through cytokine release can limit or result in fluctuating expression levels of the gene of interest^42–45^. Expression of proteins from IVT mRNA is independent of promoter activity and potential DNA modification or silencing.

As an alternative to transient overexpression, stable transfection or transduction of certain genes into cell lines are used. Depending on the cell type, establishment of such lines can be time consuming and technically demanding. This holds particularly true for hepatic cells, used to study the HBV life cycle^10^. In addition, cell lines created by selection of clones may bias analyses due to clonal differences. In addition, random genomic integration of expression cassettes, by lentiviral or plasmid-based systems, can disturb cellular gene expression and physiology.

Introduction of NTCP in HeLa, HEK293, A549 and U2OS did not allow productive HBV infection, even when NTCP was expressed at high levels supporting previous findings^46,47^. Although transfection of IVT mRNA resulted in surface expression of functional NTCP, which was confirmed by bile acid uptake and MyrB binding, this was not sufficient to render these cells permissive for HBV indicating that other host factors necessary to support HBV infection are missing^47^. The finding that human delta virus (HDV) can replicate in NTCP expressing HeLa cells^46^, indicates that NTCP allows entry into the cytoplasm but additional, cellular factors are required for subsequent replication steps of HBV. One should repeat HDV infection experiment in NTCP transfected non-hepatic cells to determine if this would render them permissive for HDV. Yang et al*.* reported that infection of HEK293 cells with HBV requires the introduction of NTCP and at least three additional factors (HNF4a, PPARa and RxRa)^48^ while this remains unknown for other, harder to transfect cell types. Using IVT mRNA may help to overcome this problem because it allows highly efficient introduction of multiple proteins simultaneously^26^ or consecutively.

We showed that IVT mRNA allows linear titration and dose-dependent protein expression in target cells achieving or even exceeding physiological expression levels. In our study, bile acid uptake as well as HBV uptake and infection mediated by NTCP was strictly dose-dependent. Our data also indicate that a certain threshold of NTCP surface expression is required to promote HBV entry and infection, and that infection efficiency at least in cell culture depends on the concentration of available NTCP. However, we demonstrate that NTCP expression exceeding a certain threshold does not further increase bile acid uptake or HBV infection, indicating for additional cellular limitations independent of the receptor. In further studies, one should quantify NTCP molecules at the plasma-membrane to determine the precise number of proteins necessary for infection e.g. by fluorescence spectrometry^32^. Such analysis could be highly demanding if at all feasible since certain viral markers like cccDNA have a low abundance hampering detection with current methods. Furthermore, in vitro infection rates are highly inefficient^32^ further impairing a clear read-out of needed NTCP threshold, especially when expressed in very low frequency on hepatocytes.. Unfortunately, application of IVT mRNA also faces problems, due to the short time span of protein expression, which may render long-term pathway analyses inefficient^30^. One option to overcome this limitation is the repeated administration of IVT mRNA to ensure steady expression of a protein of interest. Alternatively, IVT mRNA allows introducing several components of a pathways through application of multiple mRNAs simultaneously^33^. This was shown to be a suitable alternative for reprogramming fibroblasts into induced pluripotent stem cells^26^.

Taken together, we demonstrated that using IVT mRNA to introduce factors required for HBV infection is an efficient approach to decipher essential pathways and host factors. It allows efficient transfection of differentiated hepatoma cells and can also be applied for primary hepatocytes. In addition, IVT mRNA can be titrated to achieve physiological protein levels, as detected in the liver, helping to identify further liver specific host factors involved in HBV fusion, nuclear transport or rc- to cccDNA conversion. A combination of our IVT mRNA system with other tools, like cDNA libraries encoding for liver specific factors, might be a fast and efficient way to identify these unknown factors in future experiments.

## Methods

### Cell culture

HEK293 (ATCC^®^ CRL-1573™), A549 (ATCC^®^ CCL-185™), HeLa (ATCC^®^ CCL-2™), U2OS (ATCC^®^ HTB-96), HepG2 (ATCC^®^ HB-8065) and HepG2-NTCP-K7 [10] cell lines were maintained in Dulbecco’s Modified Eagle Medium: Nutrient Mixture F-12 (DMEM/F-12, Gibco, Carlsbad, USA) supplemented with 10% heat-inactivated fetal calf serum (FCS; Gibco) and 1% penicillin/streptomycin (10.000 U/mL, Gibco) in a humidified 37 °C, 5% CO_2_ incubator. HepaRG cell line (RRID:CVCL_9720) was cultivated in William’s E medium supplemented with 10% FBS Fetalclone II (Thermo Fisher Scientific, Waltham, MA, USA), 1% penicillin/streptomycin, 2 mM glutamine, 0.023 U/mL human insulin, 0.0047 mg/mL hydrocortisone and 0.08/mL gentamicin (Gibco). HepG2, HepG2-NTCP-K7 and HepaRG cells were cultivated on collagen-coated plates, HEK293 cell line on poly-l-lysine coated plates. To differentiate HepG2 and HepG2-NTCP-K7 cells, 2.5% DMSO was added to the cell culture medium for 3 days when cells were sub confluent. For differentiation of HepaRG, cells were first cultivated for 2 weeks in standard medium, followed by cultivation in medium supplemented with 1.8% DMSO for another 2 weeks. All experiments were performed in 24 well plates using 500 µL of standard cultivation medium per well.

### Cloning and mRNA production

NTCP cDNA was synthesized by reverse transcription of mRNA isolated from differentiated HepaRG cells. The open reading frame of NTCP was amplified by PCR, supplied with a GSGS-linker, fused to tdTomato and inserted into pcDNA3.1 backbone under control of either CMV or T7 promotors. tdTomato-N1 was a gift from Michael Davidson & Nathan Shaner & Roger Tsien (Addgene plasmid # 54642). For mRNA production both plasmids (pDNA-NTCP, pDNA-NTCP-tdTomato) were linearized downstream of the coding sequence with *EcoRI* (Thermo Fisher Scientific) for 1 h at 37 °C and precipitated overnight at − 20 °C using 1:20 0.5 M EDTA, 1:10 5 M NHaO2 and 1:1 100% ethanol. After washing with 100% ethanol, 1 µg of linearized plasmid was used for mRNA synthesis using HiScribe ARCA T7 in vitro transcription kit (New England Biolabs, Ipswich, MA, USA) supplemented with 10 µM ψ-UTP and 10 µM m^5^CTP (Jena Bioscience, Jena, Germany) nucleotide analogs as indicated. Thereby, production of IVT mRNA was subdivided in 2 steps: (1) ARCA capping and synthesis of IVT mRNA and (2) a subsequent addition of a poly-A tail. Synthesized IVT mRNA was analyzed on 2% agarose gel to validate correct length and tailing efficiency.

### Transfection and transduction

For mRNA and poly I:C transfection, Lipofectamine Messenger Max (Invitrogen, Carlsbad, CA, USA) was used in 1:3 ratio. pDNA transfection was performed with Lipofectamine 2000 (Invitrogen) in 1:2 ratio. mRNA and Lipofectamine incubated for 10 min, plasmid and Lipofectamine for 5 min at room temperature. DNA and mRNA lipoplexes were added to cells, cultivated in standard cultivation medium. To produce recombinant Ad-NTCP-tdTomato, the NTCP-tdTomato expression cassette was inserted into the pEntry plasmid, under control of a transthyretin receptor promoter, and recombined into an Ad5 genome, using the Gateway™ system (Thermo Fisher Scientific). The resulting pAd-NTCP-tdTomato plasmid was linearized with *PacI* and transfected in HEK293 cells. Four to 6 days post transfection, recombinant adenoviruses were harvested, using multiple freeze–thaw steps, and propagated by subsequent transduction into fresh 293 cells, before final harvest. Ad-NTCP-tdTomato was titrated on 293 cells by immunofluorescence to determine infectious units (IU) and added to cell culture medium to obtain a desired multiplicity of infection (MOI). One day post transfection or transduction medium was replaced with fresh cultivation medium. Cell viability was determined using the Cell Titer Blue assay (Promega, Madison, WI, USA) according to manufacturer’s instruction using 1:5 dilution of the reagent in standard cultivation medium. Fluorescence measurement (560(20)Ex/590(10)Em) was performed using M200 Infinite platereader (Tecan, Männedorf, Switzerland).

### Western blot

Protein lysates were obtained as previously described^5^. To remove glycosyl residues, protein lysates were treated with peptide N-glycosidase (New England Biolabs). Proteins were separated by SDS-PAGE and NTCP expression was analyzed by Western blot using rabbit antiserum K9 (kindly provided by B. Stieger) and goat anti-rabbit-HRP (Sigma-Aldrich, St. Louis, MO, USA) for detection. β-Actin was detected using mouse anti-β-Actin and goat anti-mouse-HRP (both Sigma-Aldrich). Images were obtained using Chemostar software (Intas Science Imaging, Göttlingen, Germany) and were further processed using Image J (National Institutes of Health (NIH), Bethesda, MD, USA).

### Radioactive bile acid uptake assay

Analysis of [^3^H] taurocholate uptake was performed as previously described^49^. Briefly, negative samples were incubated for 15 min at 37 °C with 200 nM MyrB diluted in 250 µL basal medium. Next, Hot Mix stock (1940 µL basal medium, 66 µL 15 mM cold TC (Sigma-Aldrich) and 1 µL hot TC (Hartmann Analytic, Braunschweig, Germany) was diluted 1:10 in basal medium, 25 µL were added to the preincubated samples and incubated for 15 min at 37 °C. After incubation, cells were placed on ice, Hot Mix was removed and cells were washed 3 times with ice-cold PBS. Next, 500 µL lysis buffer (0.05% SDS, 0.25 mM NaOH) were added and lysed cells were transferred to scintillation vials. Afterwards, 4 mL scintillation liquid was added, vials were closed and vortexed for 30 s. Measurement of [^3^H] taurocholate was performed with scintillation analyzer.

### Flow cytometry and fluorescence microscopy

For analysis of NTCP expression, 200 nM Atto_488_ labeled Myrcludex B (MyrB_Atto488_) was added to culture medium and incubated for 30 min at 37 °C. Unbound MyrB_Atto488_ was removed by washing with PBS. For flow cytometry analysis, cells were detached with trypsin, resuspended in PBS supplemented with 1% FCS and 1 mM EDTA and washed 3 × times with PBS. For HBV core staining cells were treated using BD Cytofix/Cytoperm Fixation/Permeablization Kit (Becton Dickinson, Franklin Lakes, NJ, USA) according to manufacturer’s instructions using Hepatitis B Virus Core Antigen Rabbit Polyclonal Antibody (1:1000, Cell Marque, Rocklin, CA, USA) and PE F(ab’)2 Donkey anti-rabbit IgG (1:10, #558416, Becton Dickinson) Analysis of MyrB_Atto488_ staining, NTCP-tdTomato and HBV core expression was performed with fluorescence microscope Leica DM8i (Leica Biosystems, Wetzlar, Germany) or flow cytometry (Cytoflex, Beckman Coulter, Brea, CA, USA). Fluorescence images were obtained using Leica Application Suite X (LAS X, Leica, Wetzlar, Germany). Data analysis of flow cytometry data was performed using FlowJo, LLC (Becton Dickinson).

### Quantification of various factors by qPCR

Nucleospin Tissue Kit was utilized for total cellular DNA extraction (Macherey&Nagel, Düren, Germany) according to manufacturer’s instructions. Total intracellular HBV DNA (primers: HBV1745 GTTGCCCGTTTGTCCTCTAATTC; HBV1844 GGAGGGATACATAGAGGTTCC-TTGA) and human prion protein *PRNP* (primers: PRNPfwd TGCTGGGAAGTGCCATGAG; PRNPrev CGGTGCATGTTTTCACGATAGTA) as a reference gene were amplified by qPCR on a LightCycler 480 system using SYBR Green I Master (Roche, Mannheim, Germany). For selective cccDNA detection (primers: ccc2251− AGCTGAGGCGGTATCTA; ccc92+ GCCTATTGATTGGAAAG-TATGT), samples were pretreated with T5 exonuclease (New England Biolabs) digestion. Cycling programs were used as described previously^50^. For total RNA isolation Nucleospin RNA extraction kit was used (Macherey&Nagel) and cDNA synthesis was performed using Superscript III First-Strand Synthesis SuperMix (Invitrogen) according to manufacturer’s instructions. Quantification of IFNβ (primers: IFNβfwd TTCAGTGTCAGAAGCTCCTGTGG; IFNβrev CTGCTTAATCTCCTCAGGGATGTCA) was performed relative to 18S ribosomal RNA (primers: 18Sfwd AAACGGCTACCACATCCAAG; 18Srev CCTCCAATGGATCCTCGTTA) using previously described protocol^33^.

### HBV uptake and infection

HBV uptake assay and infection were performed as previously described^10,32^. HBV stocks were prepared by heparin column purification and subsequent sucrose gradient centrifugation as described^51^. DNA-containing, enveloped particles (virions) were determined for calculation of the MOI. Qualitative Hepatitis B e antigen (HBeAg) measurement was performed using a commercial immunoassay (BEP III, Siemens Molecular Diagnostics, Marburg, Germany). Sample/Cut-off was determined using internal Cut-off value where samples > 1 are considered as positive. Quantitative HBeAg analysis was obtained using the HBeAg Reagent Kit with HBeAg Quantitative Calibrators on the Architect™ platform (Abbott Laboratories, Chicago, IL, USA).

## Supplementary Information

Supplementary Information.
