# Supplementary material for: Concentration of Na+-taurocholate-cotransporting polypeptide expressed after in vitro-transcribed mRNA transfection determines susceptibility of hepatoma cells for hepatitis B virus
Source: Sci Rep. 2021 Oct 5;11:19799. doi: 10.1038/s41598-021-99263-3 (PMC8492621; doi:10.1038/s41598-021-99263-3)

Concentration of Na^+-taurocholate-cotransporting polypeptide expressed after in vitro-transcribed mRNA transfection determines susceptibility of hepatoma cells for hepatitis B virus

Andreas Oswald[a], Anindita Chakraborty[a], Yi Ni[b.c], Jochen M. Wettengel[a], Stephan Urban[b.c] and Ulrike Protzer[1a,d,*]

- [a] Institute of Virology, School of Medicine, Technical University of Munich / Helmholtz Zentrum München, Munich, Germany.
- [b] Department of Infectious Diseases, Molecular Virology, University Hospital Heidelberg, Heidelberg, Germany
- [c] German Center for Infection Research (DZIF), partner site Heidelberg, Heidelberg, Germany
- [d] German Center for Infection Research (DZIF), partner site Munich, Germany.

Raw data for Western Blot: Figure 2 -1

Long exposure

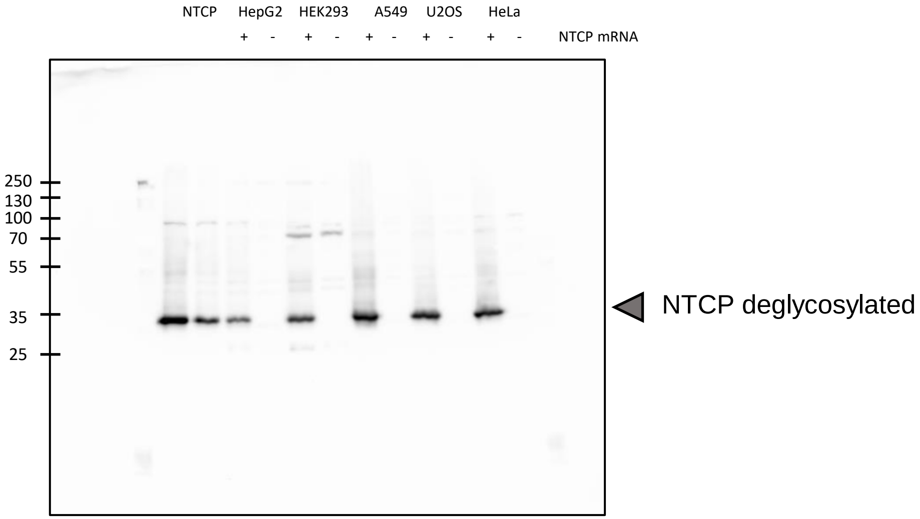

Short exposure

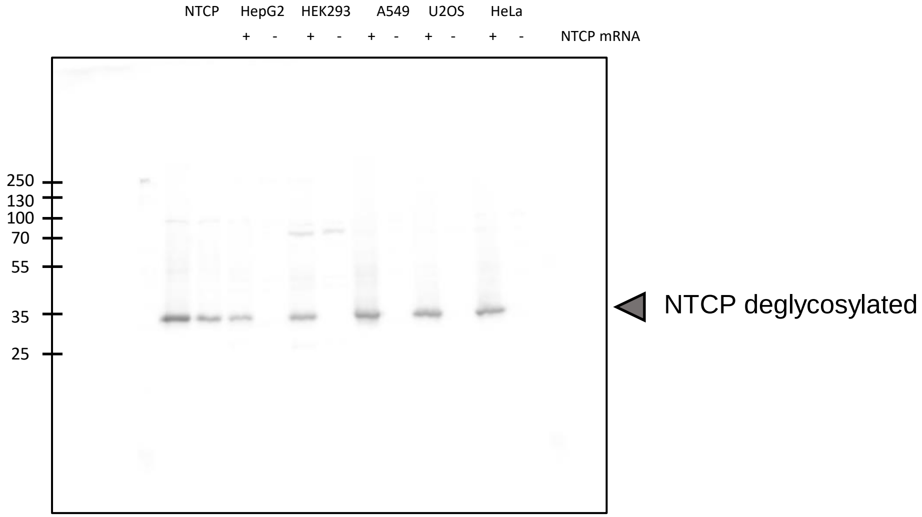

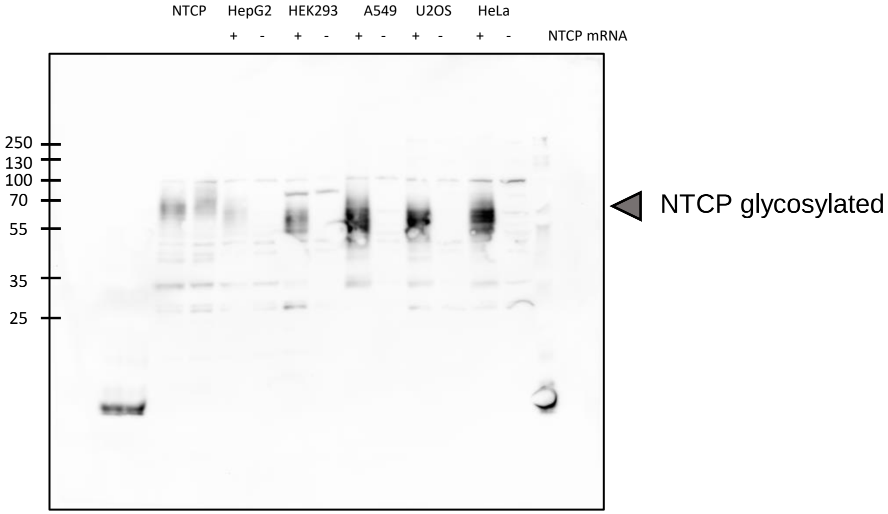

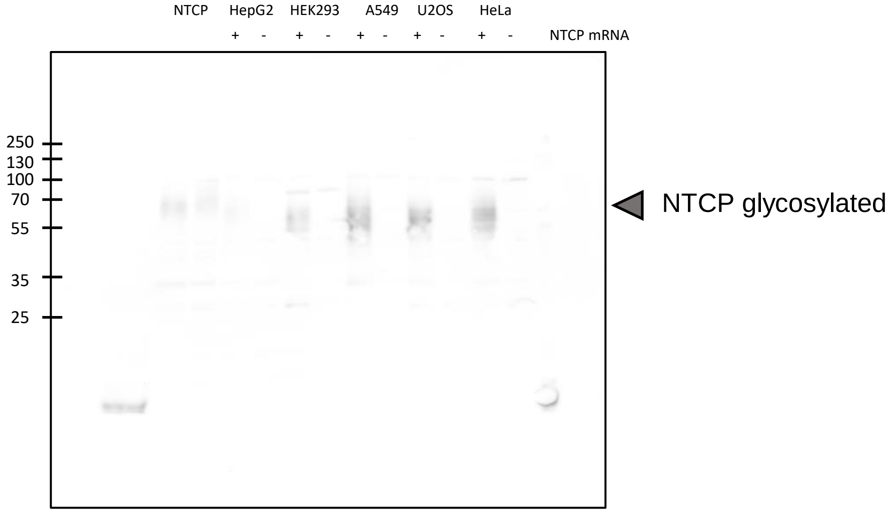

Raw data for Western Blot: Figure 2 -2

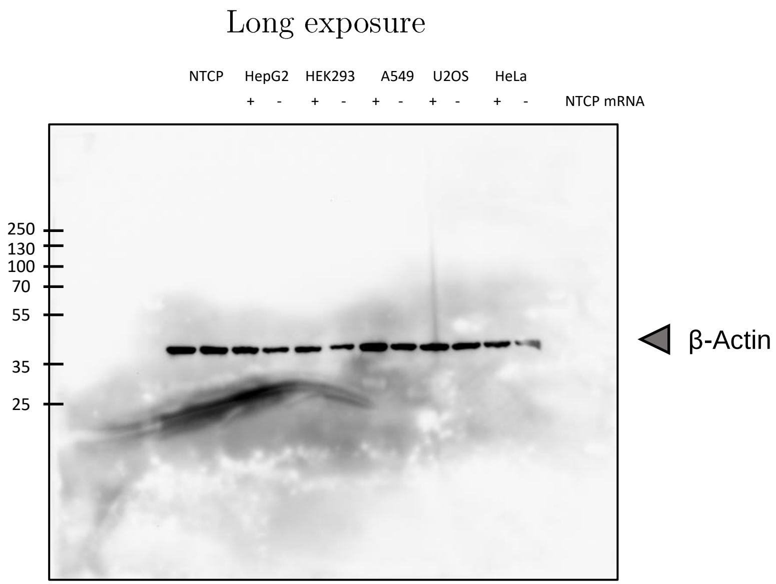

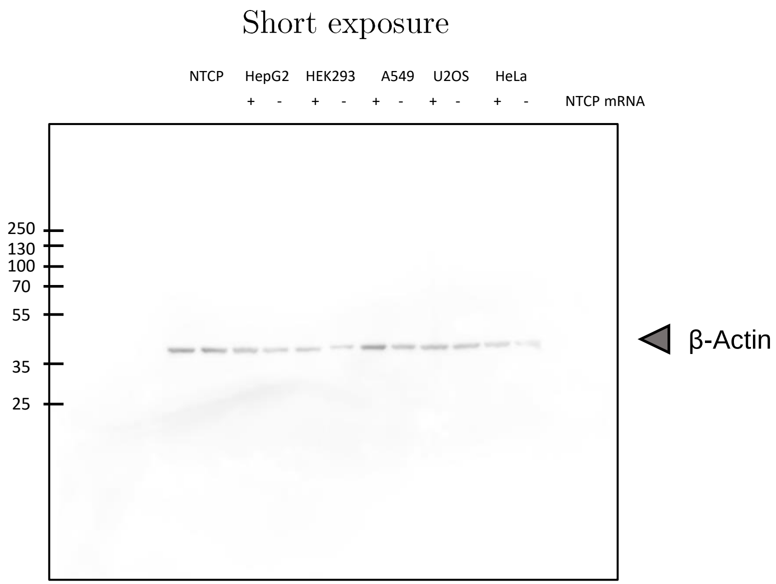

Raw data for Western Blot: Figure 5 -1

Long exposure

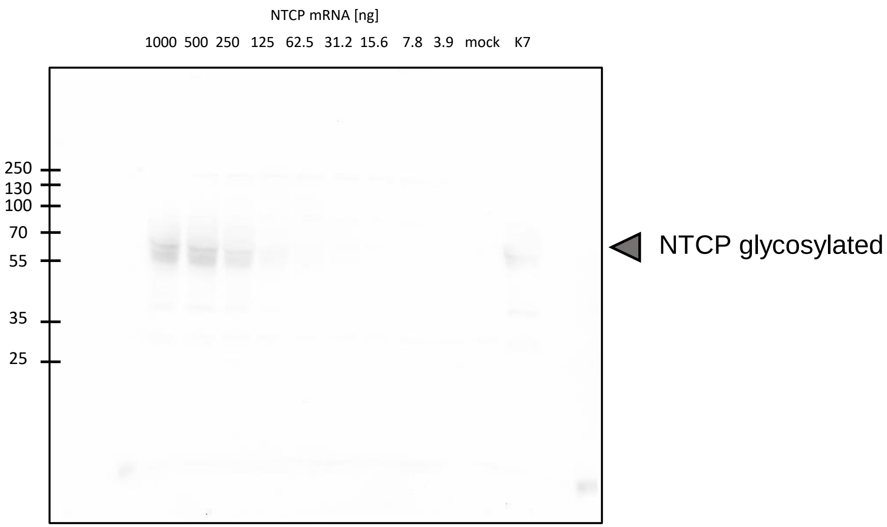

Short exposure

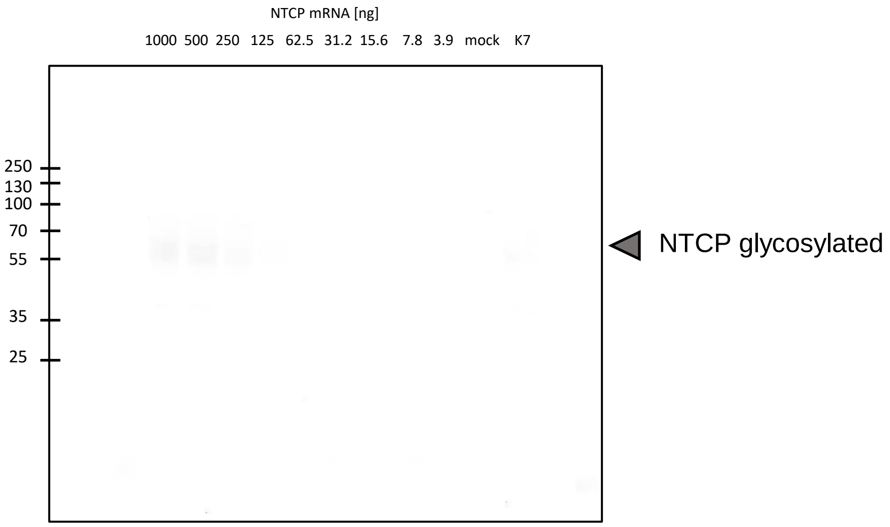

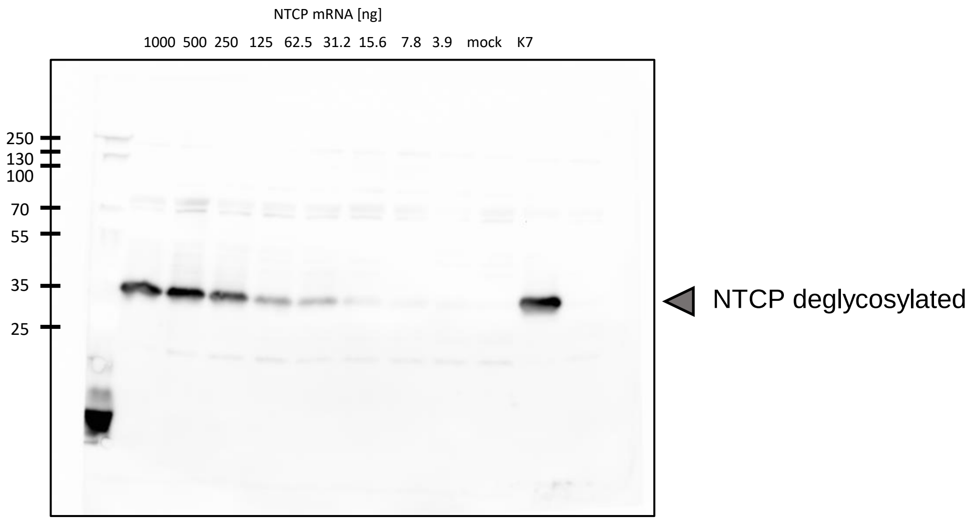

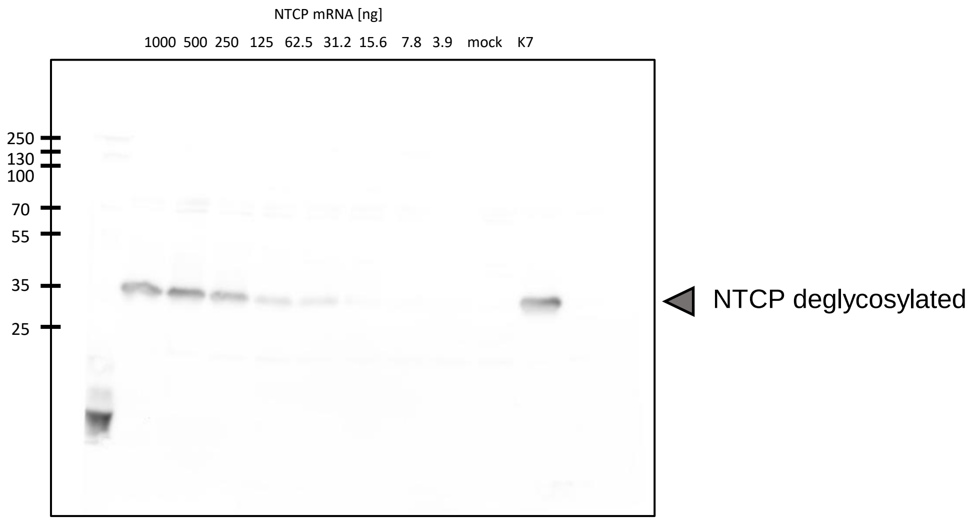

Raw data for Western Blot: Figure 5 -2

Long exposure

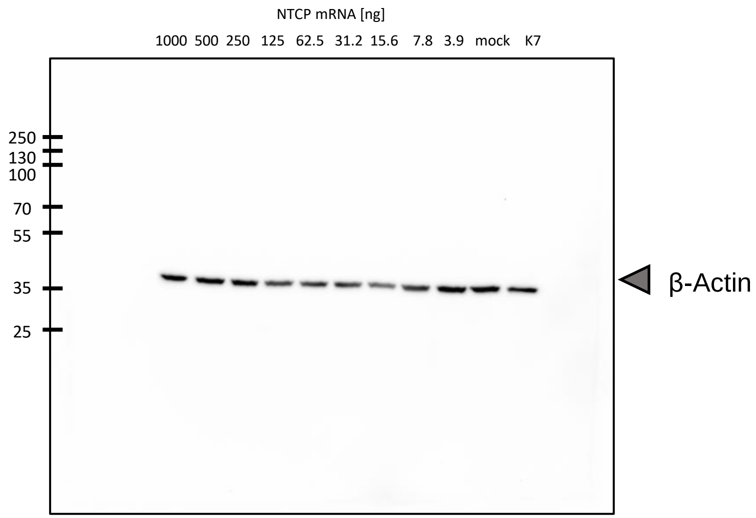

Short exposure

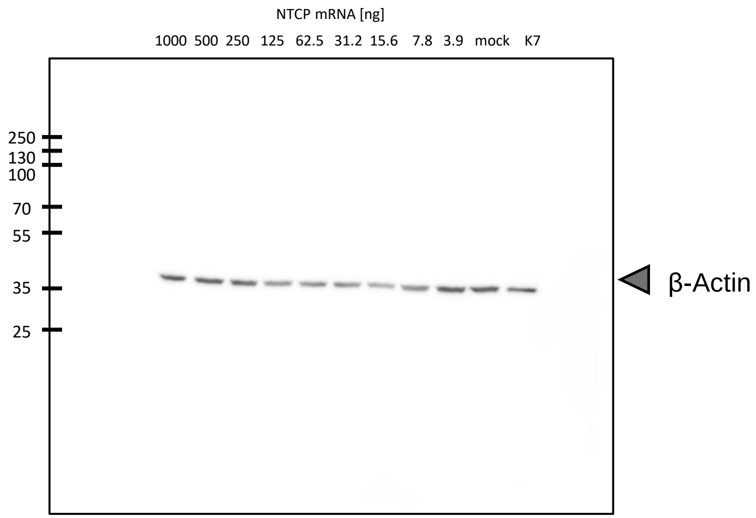

Supplement: Supplementary file 1 — Supplementary Information. [file 41598_2021_99263_MOESM1_ESM.pdf]
